# Pseudomyxoma Peritonei Arising From a Mucinous Ovarian Borderline Tumor Treated With Paclitaxel, Cisplatin, and Bevacizumab: A Case Report

**DOI:** 10.7759/cureus.67554

**Published:** 2024-08-23

**Authors:** Kentaro Niwa, Kenji Niwa, Masanori Isobe, Rui Kyogoku, Takuji Tanaka

**Affiliations:** 1 Department of Obstetrics and Gynecology, Central Japan International Medical Center, Minokamo, JPN; 2 Department of Obstetrics and Gynecology, Gujo City Hospital, Gujo, JPN; 3 Department of Obstetrics and Gynecology, Gifu University School of Medicine, Gifu, JPN; 4 Department of Diagnostic Pathology, Gifu Municipal Hospital, Gifu, JPN

**Keywords:** kras mutation, mucinous borderline tumor, ovary, laparoscopic surgery, pseudomyxoma peritonei

## Abstract

Pseudomyxoma peritonei (PMP) is a rare disease caused by primary mucinous neoplasms. Here, we describe a case where a large ovarian tumor was initially removed laparoscopically, followed by an appendectomy. The patient was diagnosed with PMP arising from an ovarian mucinous borderline tumor with a *KRAS* mutation. Treatment included bevacizumab-containing chemotherapy, resulting in complete remission.

## Introduction

Pseudomyxoma peritonei (PMP) is a rare disease characterized by loculated gelatinous ascites and peritoneal dissemination of implants [1], predominantly originating from the appendix and rarely from mucinous ovarian tumors [2]. The estimated incidence of PMP is one to two per one million people [3], suggesting that ovarian-originating PMP may occur in as few as two to four per 100 million people [4].

Historically, PMPs have been classified into three pathological subtypes: disseminated peritoneal adenomucinosis, peritoneal mucinous carcinomatosis, and an intermediate group [5]. Recently, the Peritoneal Surface Oncology Group International (PSOGI) proposed new consensus criteria categorizing PMPs based on acellular mucin, low-grade mucinous carcinoma peritonei (LGMC), high-grade mucinous carcinoma peritonei, and high-grade mucinous carcinoma with signet ring cell types, which are relevant mainly for PMPs of gastrointestinal origin [6].

In the present case, the primary ovarian tumor was initially removed laparoscopically, followed by an appendectomy. We report a case of PMP arising from an ovarian mucinous borderline tumor with a *KRAS *mutation, which achieved complete remission following bevacizumab-containing chemotherapy.

## Case presentation

**Figure 1:**
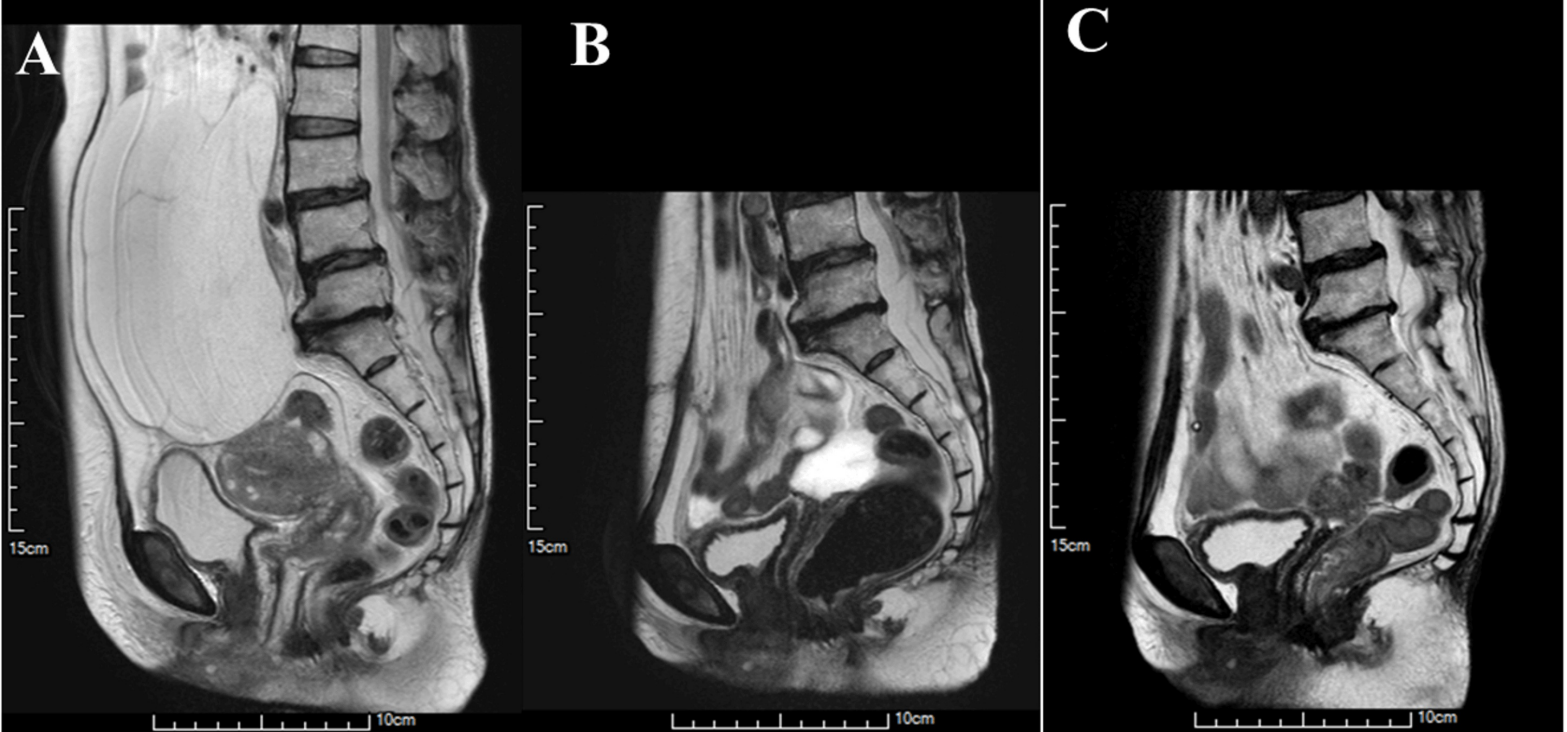
MRI images (A) before the first operation, (B) after the first operation, and (C) after the second operation. (A) Before the first operation, the MRI of the pelvis and abdomen shows a large multi-lobulated cystic mass measuring 20 cm in diameter, accompanied by somewhat viscous ascites; (B) After the first operation, the MRI reveals the presence of viscous ascites; (C) After the second operation, the MRI indicates no particular abnormalities.

A 77-year-old female patient presented with abdominal distension without pain or bowel obstructions, and sought consultation at the Department of Obstetrics & Gynecology. Ultrasound examination of the abdomen and pelvis revealed a large, multi-separated cystic mass in the right ovary accompanied by ascites. Tumor markers showed a high serum level of carcinoembryonic antigen (CEA) (19.0 ng/ml, normal range: <5.0), but serum levels of cancer antigen (CA) 125 (16.7 U/ml, normal range: <35.0) and CA19-9 (28.2 U/ml, normal range: <37.0) were within normal limits. Preoperative MRI depicted a cystic mass approximately 20 cm in diameter with internal septations, consistent with an ovarian mucinous tumor (Figure 1A). Additionally, viscous ascites and intramural uterine fibroids were observed. 

**Figure 2:**
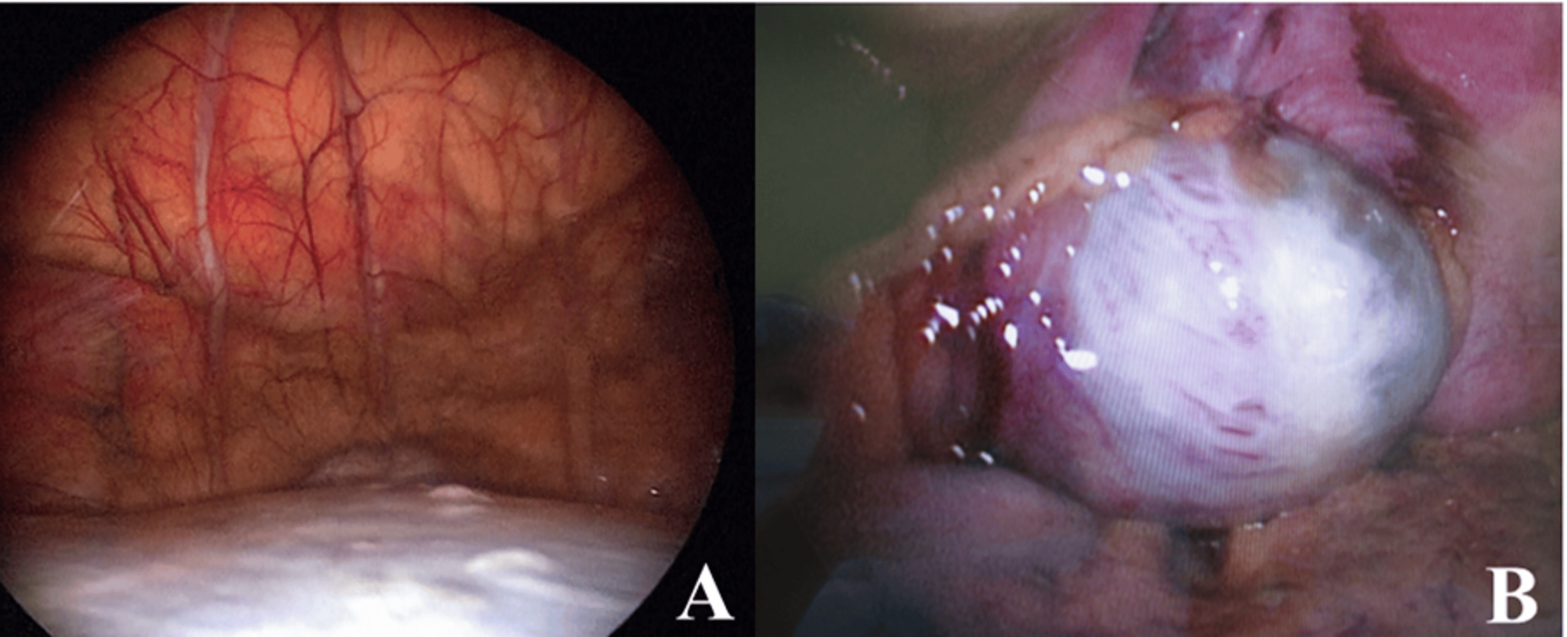
Laparoscopic views (A) before and (B) after the tumor contents were aspirated. (A) Before aspiration, the laparoscopic view shows a large cystic tumor; (B) After aspiration, the laparoscopic view shows part of the multi-cystic tumor.

**Figure 3:**
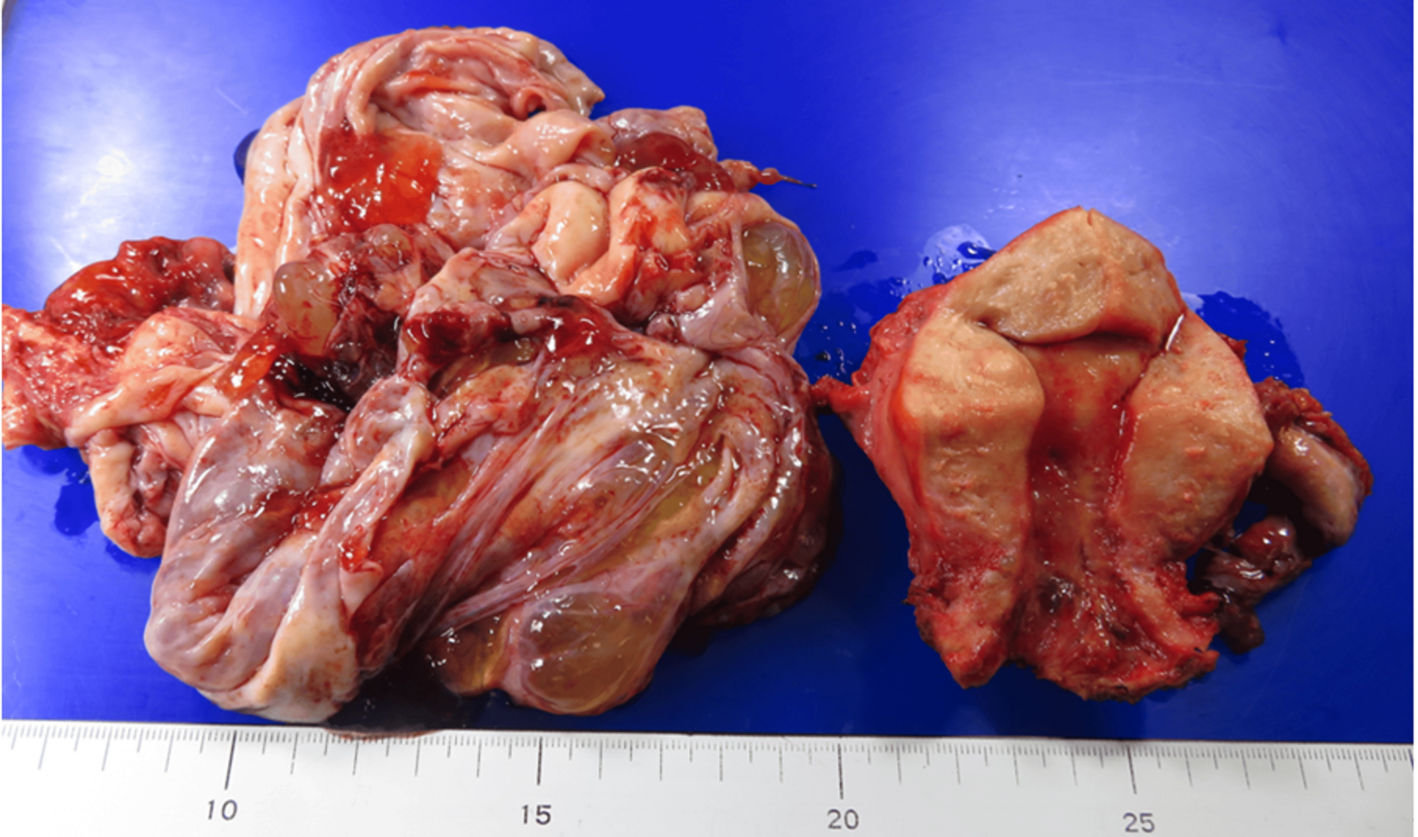
Macroscopic appearance of the excised specimen. The right ovarian tumor was multilocular and contained mucous contents. The uterus appeared to be adenomyotic, and small myomas were also found.

**Figure 4:**
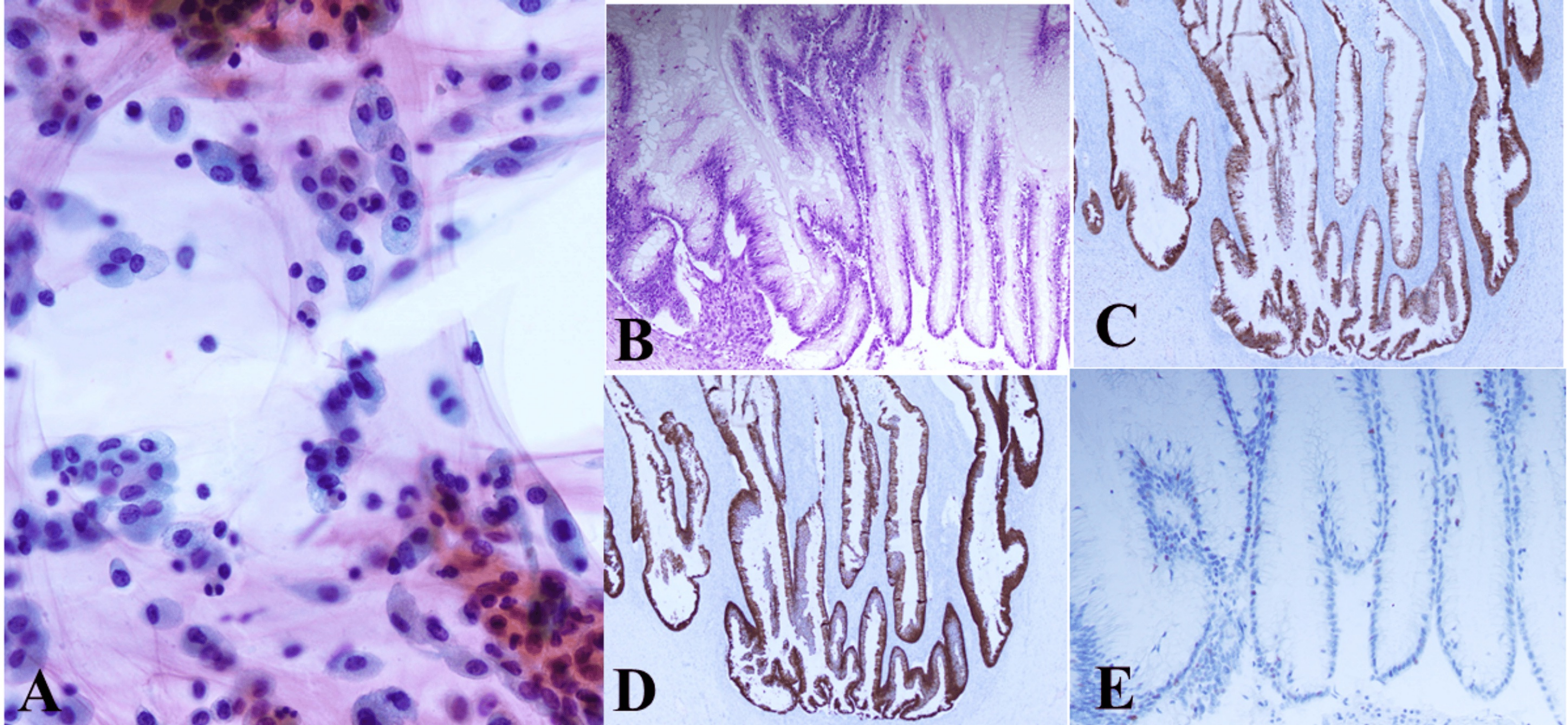
Ascitic fluid cytology (A) and histological (B) and immunohistochemical (C-E) findings of the right ovarian tumor at the time of the first surgery. (A) Cytology of the ascites shows many tumor cells suggesting mucin production, diagnosed as a mucinous borderline tumor, with slightly cellular atypia (Papanicolaou stain, x 200); (B) High columnar epithelia formed papillary structures in the cyst lumen. The tumor cells are stratified, and the tumor is diagnosed as a mucinous borderline tumor (H&E stain, x 20); (C) Immunohistochemical staining for CK7 is positive in the tumor cells (x 20); (D) Immunohistochemical staining for CK20 is also positive (x 20); (E) Immunohistochemical staining for p53 was partially and weakly positive in the tumor cell nuclei (x 20).

Approximately one month after the initial presentation, laparoscopic bilateral adnexectomy and total hysterectomy were performed with a preoperative diagnosis of benign to borderline malignant ovarian tumor and intramural uterine fibroids. During surgery, a giant cystic mass was visualized (Figure 2A). Approximately three liters of viscous fluid were aspirated from the mass without disrupting its contents (Figure 2B). Subsequently, the right ovarian tumor was resected (Figure 3), and approximately 150 ml of mucinous ascites was drained. Cytological examination of the ascitic fluid revealed mucin-producing tumor cells, consistent with a diagnosis of mucinous borderline tumor (Figure 4A). Macroscopic examination of the right ovary showed a polycystic appearance with mucus content, while the excised uterus exhibited features resembling adenomyoma and contained small fibroids (Figure 3). Pathologically, the right ovary exhibited multilayered mucinous epithelium with mild cellular atypia, confirming a diagnosis of a mucinous borderline tumor (Figure 4B). Immunohistochemical analysis showed ovarian tumor cells were positive for CK7 (Figure 4C) and CK20 (Figure 4D), and weakly positive for p53 (Figure 4E). Although macroscopic examination indicated complete tumor removal, viscous ascites persisted post surgery (Figure 1B).

**Figure 5:**
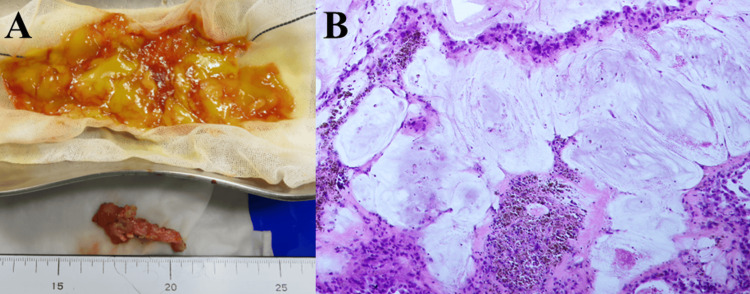
Macroscopic images of the viscous ascites and appendix removed, and microscopic finding of the resected appendix at the second surgery. (A) Very viscous yellow ascites (above) and enlarged appendix (below); (B): Tumor cells producing mucus seen in the appendix, diagnosed as mucinous cystadenoma of the appendix (x 20).

Two months later, further evaluation revealed pseudomyxoma peritonei, prompting an open appendectomy to investigate a possible primary appendix origin. Approximately 200 ml of viscous ascites was drained during the procedure (Figure 5A). Peritoneal lavage with warm low molecular weight dextran solution was performed, along with removal of microscopic retroperitoneal metastases and placement of a port for chemotherapy administration. Pathological examination identified a mucinous cystadenoma of the appendix (Figure 5B).

Intraoperative assessment using the peritoneal cancer index (PCI) [7] revealed tiny metastatic lesions in the retroperitoneum, yielding a PCI score of 3. The completeness of the cytoreduction (CCR) score [8] was determined to be 0, indicating residual disease.

*KRAS*, *NRAS*, and *BRAF* mutations in DNA extracted from formalin-fixed paraffin-embedded tissue sections of both ovarian and appendiceal tumors were analyzed at LSI Medience Corporation (Tokyo, Japan) using the MEBGEN RASKET™-B Kit (MBL Medical and Biological Laboratories Co. Ltd, Nagano, Japan), based on the method reported [9,10]. Only the *KRAS* G12V mutation was detected in the right ovarian tumor (Table 1).

**Table 1:** Resuts of KRAS, NRAS, and BRAF mutations of the right ovarian and appendiceal tumor.

RAS/BRAF	Right ovarian tumor	Appendiceal tumor
RAS pan	mutation positive	(-)
KRAS12	G12V	
KRAS13	(-)	
KRAS59	(-)	
KRAS61	(-)	
KRAS117	(-)	
KRAS146	(-)	
NRAS12	(-)	
NRAS13	(-)	
NRAS59	(-)	
NRAS61	(-)	
NRAS117	(-)	
NRAS146	(-)	
BRAF pan	(-)	(-)

The final pathological diagnosis was PMP originating from an ovarian mucinous borderline tumor staged as pT1CNxMx, with *KRAS* G12V mutation. According to PSOGI classification [6], it was categorized as LGMC. The PCI score [7] was 3, and the CCR score [8] was 0.

Postoperatively, the patient received a total of six cycles of adjuvant chemotherapy, including systemic paclitaxel and bevacizumab, along with intraperitoneal cisplatin to prevent recurrence. As of two years and four months after the second surgery, there has been no evidence of recurrence (Figure 1C).

## Discussion

Distinguishing between PMP associated with ovarian invasion and primary ovarian tumors can be challenging due to similarities between appendiceal tumors and borderline mucinous ovarian tumors [11]. Previous studies focusing on PMP originating from the appendix have identified *GNAS* and *KRAS* mutations as prominent driver genes with high mutation frequencies [12,13]. In contrast, ovarian-derived PMP is less commonly associated with *GNAS* or *KRAS* mutations but may involve mutations in other driver genes such as p53, protein tyrosine phosphatase receptor type K (*PTPRK*), and *DICER1* [4]. In this case, pathological examination identified the ovarian tumor as a mucinous borderline tumor, while the appendix exhibited a mucinous adenoma. Genetic analysis specifically detected the *KRAS* G12V mutation and tumor cells were partially positive for p53 in the ovarian tumor, although examinations for *PTPRK* and *DICER1* gene mutations were not performed.

Given that the *KRAS* G12V mutation was exclusively found in the ovarian tumor, the diagnosis was PMP originating from the ovary. Prognostic factors in ovarian-originating PMP include not only CCR and PCI but also the involvement of genes like *PTPRK* and tumor markers like CA19-9 [4]. In this case, while the serum level of CEA was elevated, CA19-9 remained within normal limits. As an early recurrence factor, there have been reports that cases in which the tumor has already ruptured at the time of surgery often recur early [14]. However, in this case, there was no rupture during surgery, and it is possible that the patient is living without recurrence.

Among the first treatment options for PMP is CRS combined with hyperthermic intraperitoneal chemotherapy (HIPEC) using cisplatin (CDDP) [15]. Studies have shown that laparoscopic CRS and HIPEC can lead to reduced hospital stays and fewer postoperative complications in carefully selected patients [16]. In this case, the disease was at a relatively early stage, and almost no tumor remained after the initial surgery, during which both the adnexa and the uterus were removed laparoscopically. The tumor was suspected to be primary PMP originating from an ovarian borderline tumor. To determine the tumor's primary origin the procedure, including an appendectomy, peritoneal washing with warm low molecular weight dextran solution, and the placement of a port in the abdominal cavity during the administration of anticancer drugs was performed. As a result, the primary site was not the appendix, and the second surgery was performed. Anti-angiogenic agents have been reported to be effective against PMP [17]. Although this case was at an early stage, a *KRAS* mutation was noted. Consequently, the postoperative chemotherapy regimen included systemic administration of paclitaxel and bevacizumab, along with intraperitoneal administration of cisplatin. Twenty-eight months after the second surgery, the patient is healthy without signs of recurrence, indicating a successful treatment outcome.

## Conclusions

We reported a case of PMP arising from an ovarian mucinous borderline tumor with a *KRAS* mutation, which achieved complete remission with a bevacizumab-containing chemotherapy regimen. This case highlights the potential for successful outcomes in PMP when treated with a combination of surgical and targeted chemotherapeutic approaches. There has been no recurrence for 28 months.
